# Amyloid precursor protein glycosylation is altered in the brain of patients with Alzheimer’s disease

**DOI:** 10.1186/s13195-020-00664-9

**Published:** 2020-08-12

**Authors:** Claudia P. Boix, Inmaculada Lopez-Font, Inmaculada Cuchillo-Ibañez, Javier Sáez-Valero

**Affiliations:** 1 Instituto de Neurociencias de Alicante, Universidad Miguel Hernández-CSIC, Av. Ramón y Cajal s/n, E-03550 Sant Joan d’Alacant, Spain; 2 Centro de Investigación Biomédica en Red sobre Enfermedades Neurodegenerativas (CIBERNED), Sant Joan d’Alacant, Spain; 3 Instituto de Investigación Sanitaria y Biomédica de Alicante (ISABIAL), Alicante, Spain

**Keywords:** Alzheimer’s disease, sAPPα, sAPPβ, Brain, Glycosylation

## Abstract

**Background:**

The amyloid precursor protein (APP) is a transmembrane glycoprotein that undergoes alternative proteolytic processing. Its processing through the amyloidogenic pathway originates a large sAPPβ ectodomain fragment and the β-amyloid peptide, while non-amyloidogenic processing generates sAPPα and shorter non-fibrillar fragments. Hence, measuring sAPPα and sAPPβ has been proposed as a means to identify imbalances between the amyloidogenic/non-amyloidogenic pathways in the brain of Alzheimer’s disease (AD) patients. However, to date, no consistent changes in these proteolytic fragments have been identified in either the brain or cerebrospinal fluid of AD individuals.

**Methods:**

In frontal cortex homogenates from AD patients (*n* = 7) and non-demented controls (NDC; *n* = 7), the expression of total APP mRNA and that of the APP isoforms generated by alternative splicing, APP695 and APP containing the Kunitz protease inhibitor (KPI), was analyzed by *q*RT-PCR using TaqMan and SYBR Green probes. The balance between the amyloidogenic/non-amyloidogenic pathways was examined in western blots estimating the sAPPα and sAPPβ fragments and their membrane-tethered C-terminal fragments CTFα and CTFβ. CHO-PS70 cells, stably over-expressing wild-type human APP, served to evaluate whether Aβ42 peptide treatment results in altered APP glycosylation. We determined the glycosylation pattern of sAPPα and sAPPβ in brain extracts and CHO-PS70 culture media by lectin-binding assays.

**Results:**

In the cortex of AD patients, we detected an increase in total APP mRNA relative to the controls, due to an increase in both the APP695 and APP-KPI variants. However, the sAPPα or sAPPβ protein levels remained unchanged, as did those of CTFα and CTFβ. We studied the glycosylation of the brain sAPPα and sAPPβ using lectins and pan-specific antibodies to discriminate between the fragments originated from neuronal APP695 and glial/KPI variants. Lectin binding identified differences in the glycosylation of sAPPβ species derived from the APP695 and APP-KPI variants, probably reflecting their distinct cellular origin. Moreover, the lectin-binding pattern differed in the sAPPα and sAPPβ originated from all the variants. Finally, when the lectin-binding pattern was compared between AD and NDC groups, significant differences were evident in sAPPα glycosylation. Lectin binding of the soluble sAPPα and sAPPβ from CHO-PS70 cells were also altered in cells treated with the Aβ peptide.

**Conclusion:**

Our analysis of the lectin binding to sAPPα and sAPPβ suggests that glycosylation dictates the proteolytic pathway for APP processing. Differences between the demented and controls indicate that changes in glycosylation may influence the generation of the different APP fragments and, consequently, the pathological progression of AD.

## Introduction

The major constituent of amyloid plaques is the β-amyloid (Aβ) peptide, which is thought to be the main pathological effector of Alzheimer’s disease (AD). Aβ is a polypeptide generated by proteolytic processing of the much larger amyloid precursor protein (APP), a ubiquitous glycoprotein expressed strongly throughout the brain. APP is a type I transmembrane protein that resembles a cell surface receptor, containing a large N-terminal ectodomain, a transmembrane domain that contains part of the Aβ sequence, and a short intracellular C-terminal domain [31]. APP is processed through the successive action of enzymes known as secretases, undergoing alternative proteolytic processing (reviewed in [2]). Thus, sequential processing of APP always commences with the cleavage of the ectodomain by the secretase sheddase, which acts at residues close to the transmembrane domain to generate a large N-terminal fragment (NTF). In the amyloidogenic pathway, APP cleavage by β-secretase results in the secretion of an exclusive NTF, sAPPβ. This pathway co-exists with the so-called non-amyloidogenic pathway that is initiated by α-secretase cleavage within the Aβ domain, precluding Aβ formation and resulting in the secretion of the sAPPα NTF. Subsequently, the membrane-tethered C-terminal fragments that remain, the CTFβ (99 amino acids, the so-called C99) and CTFα (83 amino acids, C83), are cleaved at the boundaries of the lipid bilayer by γ-secretase, releasing the extracellular Aβ (amyloidogenic pathway) or a short non-amyloidogenic peptide named p3, as well as a soluble fragment known as the intracellular domain (ICD; reviewed in [43]). Alternatively, β-secretase can cleave APP at Glu11 in the Aβ sequence, generating a 89 amino acids CTFβ (C89) that will be further processed by γ-secretase to generate a N-terminally truncated Aβ11-x species [61].

The currently prevailing idea is that an excess of soluble Aβ oligomers becomes neurotoxic and can trigger the onset of AD. However, while the amount of pathological Aβ species are expected to increase in the AD brain (particularly the Aβ42), these Aβ42 peptides are rapidly trapped into plaques. As such, it is quite a challenge to monitor the soluble Aβ in cerebrospinal fluid (CSF) as a read-out of any possible disturbance to APP amyloidogenic/non-amyloidogenic processing, or of the enhanced generation of Aβ in the brain.

Estimating the amount of unprocessed full-length APP in brain homogenates is also a challenge, not least due to the fast recycling of cell-surface APP after endocytosis [60]. Indeed, APP can be constitutively cleaved by secretases, even during its maturation [43]. In this context, CSF levels of sAPPα and sAPPβ fragments have been evaluated as a tool to detect the equilibrium between the non-amyloidogenic and amyloidogenic processing of APP, although no consistent changes have as yet been reported [45, 49]. A few studies have analyzed possible alterations to the levels of sAPPα or sAPPβ in AD patient brain homogenates [37, 72], or changes in CTFα and CTFβ [47, 51, 52], yet again with inconclusive results.

Significantly, APP undergoes alternative splicing and the significance of different APP variants in particular cell types may reflect its distinct physiological roles. These variants may also have different regulatory requirements and respond differently to pathological changes, highlighting the need for them to be fully characterized. Alternative splicing generates APP transcripts of different sizes and while the primary isoform expressed in neurons is the so-called APP695 isoform (the number indicating the amino acid residues encoded), the longer APP751 variant is mainly expressed in astrocytes and other glial cells. This longer APP variant harbors an amino acid insert in its extracellular domain that is homologous to a Kunitz-type serine protease inhibitor (KPI). Another long isoform is APP770, which is widely expressed in peripheral tissues but minimally in the brain, and that also contains the KPI domain as well as an additional domain with homology to the MRC OX-2 antigen [10, 38]. Currently, the regarding APP mRNA expression in the brain of AD patients is somewhat confusing, with reports of increases [14, 46], no significant change [19], or even decreases in expression [13, 66]. This puzzling scenario can be extended to specific APP mRNA splice variants, with studies indicating different changes in mRNA encoding the APP695 [28, 30, 46] or the APP-KPI variants [50, 64].

Glycosylation plays a critical role in the trafficking and final subcellular localization of many proteins. Newly synthesized APP undergoes various post-translational modifications, including N- and O-linked glycosylation, and its sequential proteolytic processing, either through the amyloidogenic or non-amyloidogenic pathways, occurs after glycosylation [67]. Accordingly, the trafficking and final subcellular localization of APP appears to be crucial to determine the proteolytic processing of surface APP, and changes in glycosylation have been related to differences in APP processing, particularly O-glycosylation, [27, 11, 34, 41, 42].

Hence, we set out to determine whether APP mRNA expression is altered in the brain of AD patients. In addition, we characterized and determined the balance of the proteolytic sAPPα and sAPPβ, and CTFα and CTFβ, fragments in the brain. We also examined whether the patterns of sAPPα and sAPPβ glycosylation are altered in the brain of AD subjects and in Aβ-treated cells.

## Materials and methods

This study was approved by the ethics committee at the Miguel Hernandez University, and it was carried out in accordance with the Helsinki Declaration regarding research on humans. Frozen brain samples from 7 AD patients (4 females and 3 males, 81 ± 12 years of age) and 7 non-demented control cases (NDC, 4 females and 3 males: 65 ± 15 years) were obtained from the Banco de Tejidos Neurológicos, Fundación CIEN-Unidad de Investigación Proyecto Alzheimer (UIPA: Madrid, Spain), and the Biobanco en Red de la Región de Murcia (Biobanc-Mur, Murcia, Spain), both coordinated by the neuropathologist Dr. A. Rábano (Fundación CIEN-UIPA). Cases of sporadic AD were selected based on their clinical history of dementia and a neuropathological CERAD diagnosis [39], and they were categorized as stages V–VI on the Braak and Braak scale [6]. Samples from NDCs corresponded to individuals with no recognized clinical dementia and no evidence of any other brain pathology. The mean post-mortem interval of the tissue was between 1.5 and 6 h, with no significant differences between the two groups.

### Brain homogenization and extraction

Samples of human frontal cortex stored at − 80 °C were thawed gradually at 4 °C and then homogenized in ice-cold extraction buffer (10% wt/vol) supplemented with a cocktail of proteinase inhibitors (cat# P834; Sigma Aldrich): 50 mM Tris-HCl [pH 7.4], 150 mM NaCl, 0.5% Triton X-100, and 0.5% Nonidet P-40 [5]. The homogenates were sonicated and centrifuged at 100,000×*g* and 4 °C for 1 h, and the supernatant was collected, aliquoted, and frozen at − 80 °C until use. Total protein concentrations were determined using the bicinchoninic acid method (Pierce).

### APP over-expressing cells

To obtain conditioned cell-culture medium, CHO-PS70 cells stably over-expressing wild-type human APP (APP751) and the γ-secretase catalytic subunit presenilin-1 [73] were grown for 48 h in six-well plates (350,000 cells/well) in Dulbecco’s modified Eagle’s medium (DMEM) plus GlutaMAX™ (Gibco® Life Technologies, Paisley, UK), supplemented with 5% fetal bovine serum (FBS: Gibco) and 100 μg/mL penicillin/streptomycin (Gibco). To allow APP-CTFs to accumulate, the cells were treated with the γ-secretase inhibitor DAPT (5 μM, LY-374973 (N-[N-(3,5-difluorophenacetyl)-l-alanyl]-S-phenylglycine t-butyl ester: Calbiochem®, Merck KGaA), as described previously [62]. Control cells were treated with the same volume of the dimethyl sulfoxide (DMSO) vehicle alone. After an 18 h exposure to the inhibitor, the cells were washed twice with cold phosphate-buffered saline (PBS) and resuspended in 100 μL of ice-cold extraction buffer supplemented with a cocktail of protease inhibitors. The cell lysates were sonicated and centrifuged for 1 h at 70,000×*g* and 4 °C, and the extracts were frozen at − 80 °C for future analysis.

Alternatively, CHO-PS70 cells were treated with Aβ1-42 peptide (Aβ42) for glycosylation analysis by lectin binding. The Aβ1-42 peptide (Aβ42) and the scrambled control peptide (AIAEGDSHVLKEGAYMEIFDVQGHVFGGKIFRVVDLGSHNVA) (Anaspec Peptide, Eurogentec) were dissolved in sterilized distilled water at a concentration of 1 mg/mL, aliquoted, and stored at − 80 °C until use. Suspensions of Aβ42 or the scrambled peptide at a final concentration of 5 μM were added to the cells, once a day for 2 days, without changing the cell media. The conditioned medium from each culture dish was removed, centrifuged at 1000×*g* at 4 °C for 5 min, and the supernatant was recovered and stored at − 80 °C till use.

### RNA isolation and *q*RT-PCR analysis of transcripts

Total RNA was isolated from human brain cortical tissue using the TRIzol Reagent and the PureLink™ Micro-to-Midi Total RNA Purification System (Invitrogen), following the manufacturer’s instructions. First-strand cDNAs were obtained by reverse transcription of this total RNA (1.5 μg) using the High Capacity cDNA Reverse Transcription Kit (Applied Biosystems; Life Technologies Paisley, UK), according to the manufacturer’s instructions. Quantitative reverse transcription-polymerase chain reactions (*q*RT-PCRs) were performed using a StepOne-Plus™ Real-Time PCR System with the Power SYBR® Green PCR Master Mix (Applied Biosystems, Carlsbad, CA, USA), according to the manufacturer’s instructions. Primers were designed to analyze the total APP brain transcripts as a whole (forward 5′-AACCAGTGACCATCCAGAAC-3′, reverse 5′-ACTTGTCAGGAACGAGAAGG-3′), as well as the APP695 (forward 5′- GGTGGTTCGAGTTCCTACAA-3′, reverse 5′- CCTCTCTTTGGCTTTCTGGA-3′) and APP-KPI species (forward 5′- CCCGAGATCCTGTTAAACTTC-3′, reverse 5′- CCTCTCTTTGGCTTTCTGG-3′) [20]. Commercial primers were obtained for the house-keeping human 18S gene (Applied Biosystems, Carlsbad, CA, USA). In addition, *q*RT-PCR was also performed using TaqMan Gene Expression Assays to amplify the total APP transcripts (Hs00169098-m1 for APP and Hs03003631-g1 for 18S; Thermo Fisher) and the TaqMan PCR Master Mix. Transcript levels were calculated by the comparative 2^−ΔCt^ method relative to the 18S cDNA.

### Western blotting

Brain extract samples (30 μg of protein per lane) were boiled at 95 °C for 5 min and resolved by 7.5% SDS-PAGE. The APP species in the samples were detected using: a rabbit polyclonal anti-APP C-terminal antiserum (1:1000; Sigma Aldrich, St. Louis, MO, USA: referred to here as Sigma-Ct); a rat monoclonal antibody named 2D8 raised against the N-terminal domain of Aβ, thus that detects CTFβ but not CTFα (1:50, [71]), a rabbit polyclonal anti-sAPPβ antiserum specific to the C-terminus of sAPPβ (1:100; IBL, Hamburg, Germany: referred to here as IBL-β); a mouse monoclonal anti-sAPPα antibody specific to the C-terminus of sAPPα (1:100; IBL, referred to here as IBL-α); and a rabbit polyclonal anti-KPI antiserum specific to the KPI domain of APP (1:500; Millipore: referred to here as KPI). The immunoreactive signal from the APP bands was quantified in western blots of the cerebral cortex tissue. Vinculin (1:2000, anti-vinculin mouse monoclonal antibody, sc-73614 Santa Cruz; 1:1000, anti-vinculin rabbit antiserum, Sigma V4139) and GAPDH (1:10000, anti-GAPDH mouse monoclonal antibody, Proteintech 60004-1) served as loading controls. Band intensities were analyzed using LI-COR software (Image Studio Lite). Using ELISA assays, the manufacturer estimated that cross-reactivity between the sAPPα and sAPPβ fragments is less than 1.5% and that neither antibody cross-reacts with full-length APP. The specificity of these pan-specific sAPPα and sAPPβ antibodies has also tested been previously in western blots [36]. The blots were probed with the appropriately conjugated secondary antibodies (IRDye 680RD goat anti-mouse; IRDye 800CW goat anti-rabbit; IRDye 680RD goat anti-rabbit or IRDye 800CW goat anti-rat: all from LI-COR Biosciences GmbH, Bad Homburg, Germany) and analyzed on an Odyssey Clx Infrared Imaging System (LI-COR).

### Lectin binding and an analysis of enzymatic deglycosylation

Brain extracts and culture media samples were incubated overnight at 4 °C with lectins specific to terminal sugars immobilized to sepharose or agarose beads: the mannose-binding lectin Con A (from *Canavalia ensiformis*; Sigma); and the lectin that recognizes more complex type N-glycans, the biantennary galactosylated N-glycan with bisecting N-acetylglucosamine-binding lectin PHA (PHA-L lectin from *Phaseolus vulgaris*; Vector). The fraction of proteins not linked to lectins was separated by centrifugation and analyzed in western blots probed with antibodies against sAPPα and sAPPβ. The proportion of non-lectin-bound APP was calculated as the ratio of non-lectin-bound APP immunoreactivity to the total immunoreactivity, the latter obtained from an aliquot maintained under the same conditions but not incubated with a lectin. All the analyses were carried out in duplicate.

Brain glycoproteins were also assessed using an enzymatic deglycosylation kit from ProZyme (GK80110), following the manufacturer’s instructions, and analyzed in western blots. The glycoproteins were deglycosylated with N-Glycanase, O-Glycanase, and Sialidase A following a protocol for full deglycosylation of the proteins. This treatment removes all *N*-linked and simple *O*-linked glycans (including polysialylated moietes) from glycoproteins.

### Statistical analysis

All data were analyzed in SigmaStat (Version 2.0; SPSS Inc.), applying a Student’s *t* test (two-tailed) or a Mann-Whitney *U* test for determining the exact *p* values (*p* values < 0.05 were considered significant). The results are presented as the means ± SEM and the correlation between variables was assessed by linear regression analyses.

## Results

### Increased APP expression in the brain of AD subjects

**Fig. 1:**
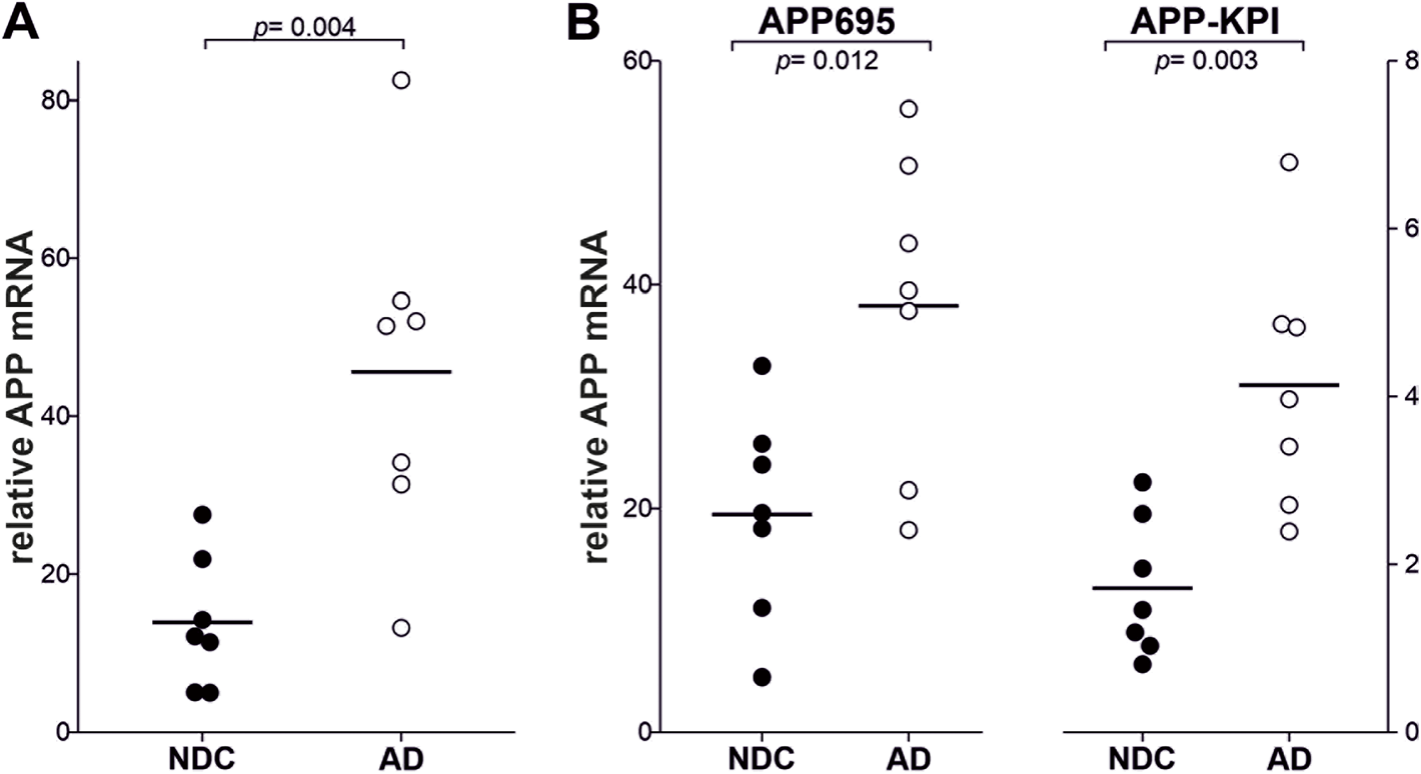
Increased APP mRNA in the frontal cortex of AD patients. Relative mRNA expression of transcripts for **a** total APP, and the **b** APP-KPI and APP-695 splice variants, analyzed by *q*RT-PCR in frontal cortex tissue from NDC (*n* = 7) and AD subjects (Braak stages V–VI, – = 7). To analyze APP transcripts, specific Power SYBR® Green PCR Master Mix primers were employed and the specificity of the PCR products was confirmed by analyzing the dissociation curves. Transcript levels were calculated by the comparative 2^−ΔCt^ method with respect to 18S rRNA from the same cDNA and expressed as the mean ± SEM: *p* < 0.001 relative to the NDCs as indicated

The expression of total APP was quantified in brain samples from AD and control subjects by *q*RT-PCR, using SYBR Green and primers designed to amplify exon 3, which is common to all APP variants. The overall expression of APP was significantly higher in the AD cortical tissue than in the NDC tissue (*p* = 0.004, Fig. 1a). The increase was corroborated when the same samples were re-analyzed using Taqman probes (exons 10–11, common to the major brain variants) for total APP (*p* < 0.001: Supplemental Fig. 1A). The APP expression assessed with the SYBR Green primers and Taqman probes was significantly correlated (*r* = 0.7, *p* = 0.006), as reported for other genes assayed with both techniques [4, 63].

To determine whether the increment in total APP mRNA in AD patients corresponds to a particular splice variant, we next analyzed APP695 and APP-KPI variants mRNA expression, using specific primers for SYBR Green. The expression of both, APP695 and APP-KPI, was significantly increased in AD brain respect to that in the control brain (Fig. 1b). This was particularly noticeable for APP-KPI mRNA (2.4 times higher in AD than in control) than for APP695 (2.0 times higher in AD than in control).

### Characterization and determination of sAPPα, sAPPβ, CTFα, and CTFβ in the brain of AD subjects

We next examined the APP proteolytic fragments, sAPPα or sAPPβ, in western blots of the brain tissue from AD subjects, a method that enabled different APP species to be discriminated, especially those with different molecular masses. However, sAPPα or sAPPβ are predicted to be only ~ 5–10 kDa smaller than full-length APP, and as such, it is not possible to distinguish these variants by electrophoretic separation [22]. Moreover, small differences in electrophoretic migration may also be attributed to differences in glycosylation [9], or even reflect immature forms of the protein [70]. Accordingly, we discriminated these sAPPα and sAPPβ variants using pan-specific antibodies generated against the specific C-terminal domain, antibodies with proven specificity [36].

**Fig. 2:**
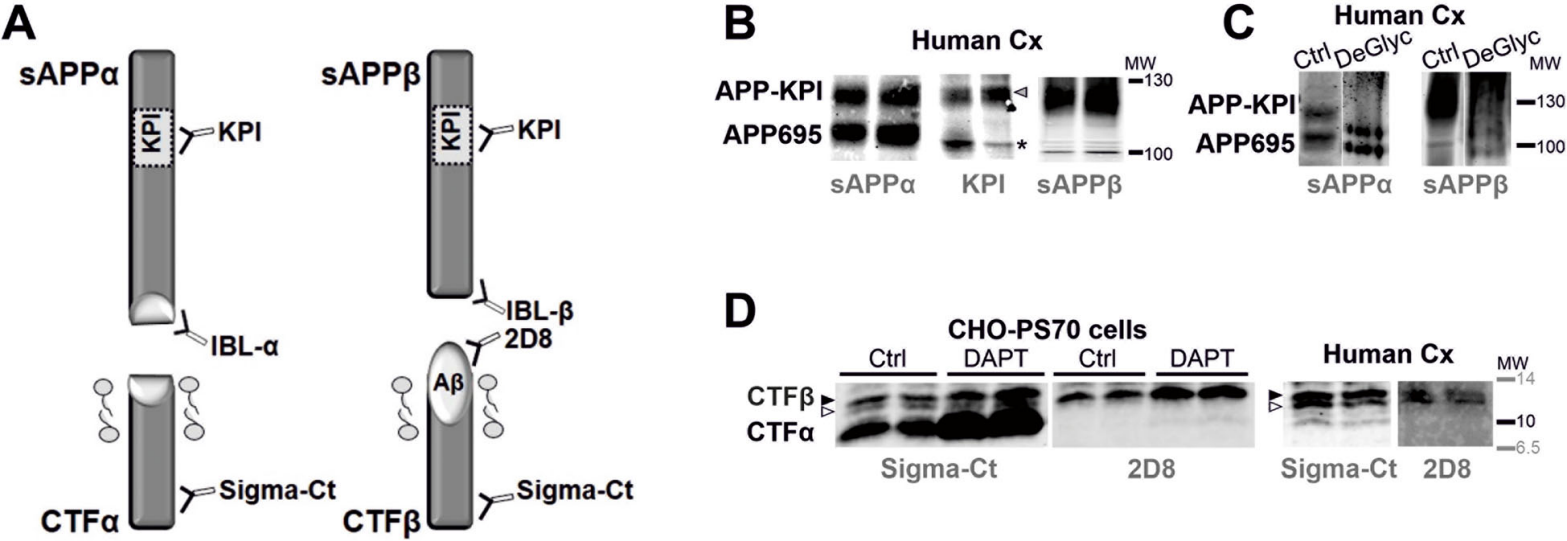
Biochemical characterization of APP-NTF and CTF fragments in frontal cortex extracts. **a** Schematic representation of the sAPPα, sAPPβ, CTFα and CTFβ proteolytic fragments generated by α-secretase (non-amyloidogenic pathway) and β-secretase (amyloidogenic pathway) (not drawn to scale). The localization of the KPI domain in APP751 and APP770 variants, but not in APP695, is indicated. The epitopes for the antibodies used in this study are also shown. **b** To assess the identity of the higher molecular mass sAPPα and sAPPβ species derived from KPI variants, the same two frontal cortex extracts were run in parallel and probed separately with an antibody against sAPPα or sAβPPβ, as well with an antibody against KPI (for a simultaneous analysis of fluorescence with the mouse anti-sAPPα antibody combined with the rabbit anti-KPI antibody: see Supplemental Fig. 2). The arrowhead indicates the KPI band that matches with the sAPPα and/or sAPPβ species, and (*) indicates the band immunoreactive for KPI but that does not match with the APP bands detected with anti-sAPPα or sAPPβ antibodies (see comments in Result section). **c** Aliquots of the frontal cortex extracts were also incubated with a cocktail of N- and O-linkage specific glycosidases, Sialidase A (deGlyc) or the vehicle alone as a control (Ctrl), and then analyzed in western blots probed with specific sAPPα or sAβPPβ antibodies (for a simultaneous analysis of fluorescence see Supplemental Fig. 2B). Representative blots are shown. **d** To characterize CTFα (C83) and CTFβ (C99 and C89), CHO-PS70 cells treated with the γ-secretase inhibitor DAPT or the vehicle alone (Ctrl) were analyzed in parallel to control brain extracts (human frontal cortex: Human Cx). Immunoblots of CHO-PS70 extracts were probed simultaneously with two different antibodies: the rabbit C-terminal antibody common to CTFα and CTFβ; and the rat antibody 2D8 raised against the N-terminal domain of Aβ that therefore only detects CTFβ (see also Supplemental Fig. 2D). The closed arrowhead indicates the specific band assigned to C99 CTFβ, which accumulates in cells treated with DAPT, while the open arrowhead indicates the band assigned to C89 CTFβ. The approximate localizations of the molecular weight (MW) markers are shown

In previous studies, APP was detected as several bands that migrate between 100 and 130 kDa [40, 59]. These differences in molecular mass possibly reflect splice variants of the predominant neuronal APP695 and the glial species that included the KPI domain that is indistinguishable in size, i.e., APP751 and APP770. All these variants are subjected to proteolytic processing by secretases and thus, they produce similar sAPPα and sAPPβ. As such, a scheme has been devised that represents the full-length APP, and the NTFs and CTFs generated, with indications of the epitopes recognized by the antibodies used in this study (Fig. 2a). The brain APP-NTF species were characterized with pan-specific antibodies for sAPPα or sAPPβ (Fig. 2b), but also with an anti-KPI antibody, indicating that only the higher molecular mass bands are sAPP species derived from KPI (see also Supplemental Fig. 2A for a multiplex assay of fluorescence). The additional band of ~ 100 kDa detected by the anti-KPI antibody might correspond to an immature APP species. We previously observed a band of similar molecular mass in human CSF when simultaneously using antibodies against KPI and the APP N-terminal [36]. Alternatively, because this band was not recognized by sAPPα and sAPPβ antibodies, it might correspond to the nearest relative of APP, the amyloid precursor-like protein 2 (APLP2), which is also regulated by alternative splicing of the KPI domain [56]. However, further analysis will be required to define the true identity of this band.

Since sAPPβ and sAPPα share the same sequence except for the last 16 C-terminal amino acids, they would not be expected to be distinguished in western blots on the basis of their size. However, when brain extracts were assessed in parallel with sAPPα- and sAPPβ-specific antibodies, differences in molecular mass were evident, particularly for the species derived from APP695 (Fig. 2b, c). These differences were corroborated when western blots were probed simultaneously with these specific sAPP antibodies using multiplex fluorescence imaging (Supplemental Fig. 2B). Conditioned media from CHO cells over-expressing APP751 (APP-KPI) also indicated that the immunoreactive bands for sAPPα and sAPPβ did not overlap (Supplemental Fig. 2C).

Treatment with N- and O-glycosidases that fully deglycosylate APP caused a reduction in the apparent molecular mass of the sAPPα and sAPPβ fragments derived from APP-KPI, while the shift of sAPPα and sAPPβ derived from APP695 was less than expected (Fig. 2c). The differences in the electrophoretic mobility between glycosylated and deglycosylated glycoproteins are probably related to the carbohydrate mass but also, to changes in protein shape that affects their migration since glycosylated proteins are normally more globular as their carbohydrate chains are not linear, even in reducing conditions. Interestingly, deglycosylated sAPPα and sAPPβ had a similar molecular mass (Fig. 2c) and even co-localized (Supplemental Fig. 2B, C), suggesting that the differences in the molecular mass observed between native sAPPα and sAPPβ species are mainly due to differences in their glycosylation.

The CTFα and CTFβ do not differ between the APP variants, and they were characterized using extracts of CHO-PS70 cells stably overexpressing wild-type human APP and PS1 [73]. Extracts of these cells treated with the γ-secretase inhibitor DAPT were probed with a C-terminal antibody, providing evidence of the accumulation of CTFα and CTFβ. These bands matched those bands found in brain homogenates loaded in parallel (Fig. 2d). When blots were probed with the rat 2D8 antibody raised against the N-terminal domain of Aβ, CTFα and CTFβ could be discriminated as the epitope recognized by this antibody is absent in CTFα (see scheme in Fig. 2a). Both antibodies resolved the doublet attributed to CTFβ, probably C99 and C89, although the upper band (C99) accumulated more in DAPT-treated cells (Fig. 2d; and Supplemental Fig. 2D for a simultaneous analysis using multiplex fluorescence imaging). Indeed, the γ-secretase inhibitor was previously seen to provoke an increase in the APP C99/C89 ratio [61], making it plausible that the lower band is APP C89.

**Fig. 3:**
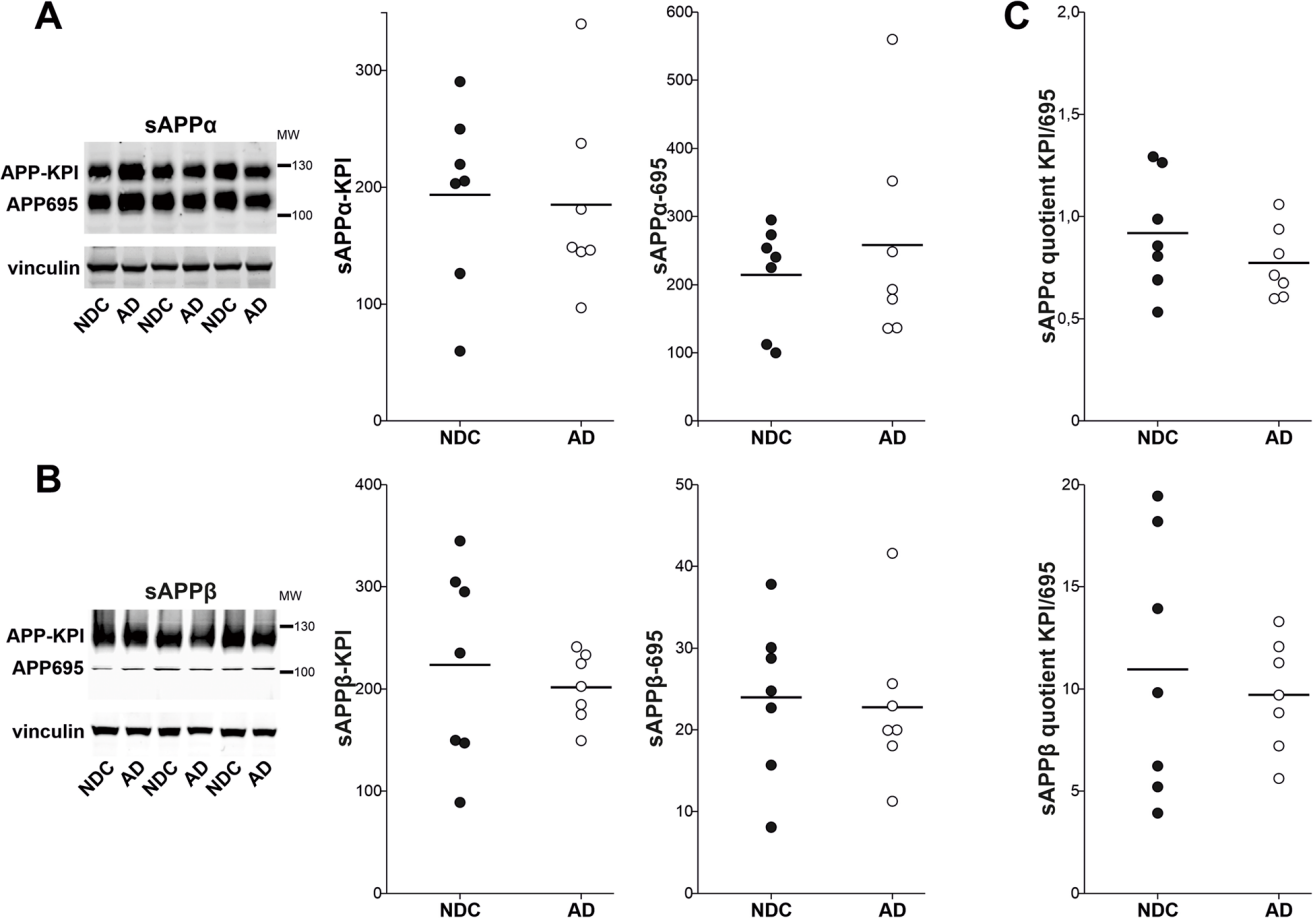
The sAPPα and sAPPβ variants remain unaltered in the AD frontal cortex. Representative western blots of human frontal cortex samples from NDC (closed symbol; *n* = 7) and AD subjects (open symbol; *n* = 7) probed with antibodies against sAPPα (**a**) or sAPPβ (**b**). The densitometric quantifications of the species attributed to the APP-695 and APP-KPI variants (see Fig. 1c) are shown, with equivalent amounts of protein loaded in each lane and using vinculin as a loading control. Calculations were performed in duplicate. **c** Graph of the quotients obtained by dividing the values for the sAPP species (sAPPα or sAPPβ) derived from the APP-KPI variant by those derived from APP695 for each sample. None of the comparisons resulted in statistically significant differences between the AD and NDC samples

After this biochemical characterization, the different sAPPα and sAPPβ isoforms were assessed in extracts from the human brain cortex. No differences were detected between the NDC and AD samples in the relative levels of sAPPα (Fig. 3a) or sAPPβ (Fig. 3b) derived from APP695 or APP-KPI. Despite the large differences in the APP695/APP-KPI ratios for the sAPPα and sAPPβ species, no differences in these ratios were evident between NDC and AD samples (Fig. 3c). The accumulation of sAPPα-695 and sAPPα-KPI immunoreactive bands was positively correlated in both NDC (*r* = 0.76; *p* = 0.048) and AD (*r* = 0.96; *p* < 0.001) tissue extracts, although this correlation was not significant for the sAPPβ species (NDC: *r* = 0.19; *p* = 0.69; AD: *r* = 0.57; *p* = 0.17). There were no significant correlations between the immunoreactivity of sAPPα and sAPPβ irrespective of whether they were derived from the APP695 or APP-KPI isoforms, or if the samples were from NDC or AD subjects (Supplemental Fig. 3).

The immunoreactive bands attributed to APP-KPI were also assessed in blots of frontal cortex extracts probed with an antibody raised against the KPI domain. No significant changes were evident in the relative levels of APP-KPI species between AD and NDC subjects (Supplemental Fig. 4). The assignation of the KPI-immunoreactive band as APP-KPI was based on its previous co-localization with the anti-sAPPα antibody (Supplemental Fig. 2A). However, the anti-KPI antibody cannot distinguish between species derived from sAPPα and sAPPβ, or between large NTF fragments and full-length variants, all these species potentially overlapping in terms of their electrophoretic mobility. Thus, our data are only indicative of APP-KPI levels in frontal cortex extracts as a whole.

**Fig. 4:**
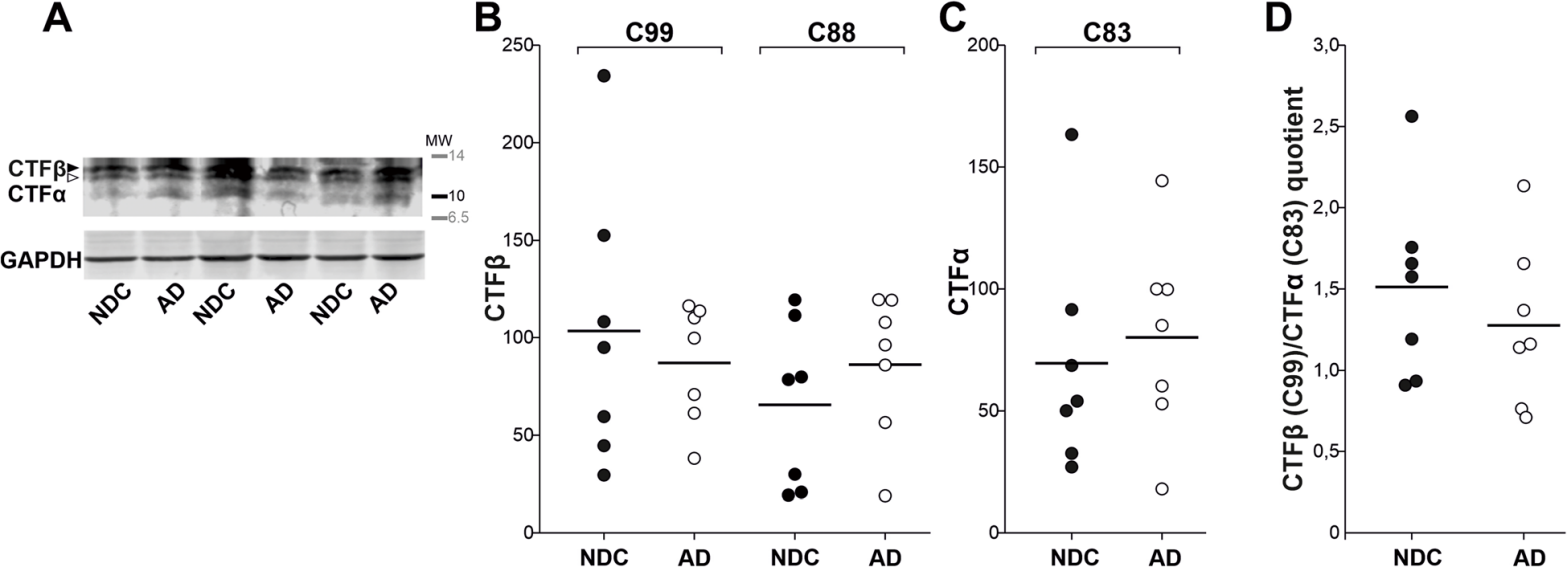
CTFα and CTFβ remain unaltered in the AD frontal cortex. **a** Representative western blots of human frontal cortex tissue from NDC (closed symbol; *n*= 7) and AD (open symbol; *n*= 7) subjects probed with a C-terminal antibody (of the full-length APP). Densitometric quantification of the **b** CTFβ species, C99 (closed arrowhead) and C88 (open arrowhead) (for characterization see Fig. 1d) or **c** CTFα (C83), is shown; using GAPDH as a loading control to ensure equivalent amounts of protein were loaded in each lane. The calculations were performed in duplicate. **d** Graph of the quotient obtained for each sample by dividing the CTFβ (C99) immunoreactivity with that for CTFα (C83), for which no statistically significant differences were evident (as was also the case for the CTF quotients not shown: C99/C89; C89/C83)

To assess whether APP-CTF levels were altered in brain extracts from AD patients, we used an antibody raised against the original C-terminal domain of full-length APP (Fig. 4a). The immunoreactivity for brain CTFβ (C99 and C89: Fig. 4b) and CTFα (Fig. 4c) was similar between NDC and AD subjects. Moreover, the CTFβ (C99)/CTFα (C83) ratio did not indicate differences between the AD and NDC groups (Fig. 4d).

### Lectin-binding analysis of sAPPα and sAPPβ from brain and Aβ-treated cells

**Fig. 5:**
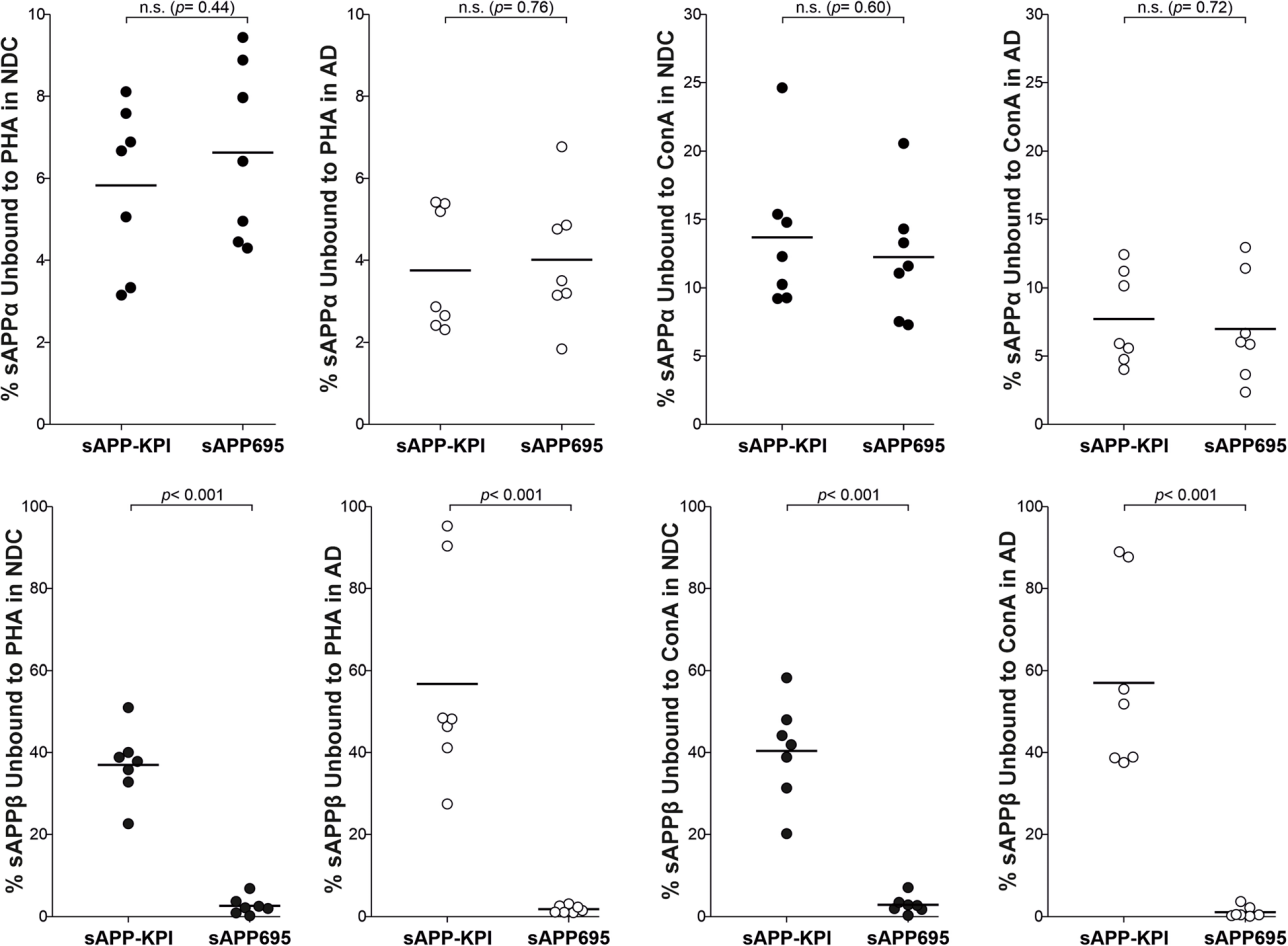
Comparison of the APP695 and APP-KPI glycosylation in brain extracts from NDC and AD subjects. Graphical representation of the relative (%) sAPPα and sAPPβ species derived from APP695 or APP-KPI variants from the 7 NDC and 7 AD brain extracts that did not bind to the immobilized PHA and Con A lectins, as indicated in Table 1. The exact *p* values are indicated (n.s., not significant)

To compare the pattern of brain sAPPα and sAPPβ glycosylation, aliquots of the brain sample extracts were incubated with immobilized Con A and PHA lectins, and sAPPα and sAPPβ pan-specific antibodies were used to probe the unbound fraction in western blots (Table 1). Since a different cell origin was presumed, we expected to detect differences in the binding properties of the APP-NTF fragments derived from the distinct splice variants. The sAPPβ species derived from APP695 and APP-KPI displayed differences in binding to both lectins, Con A and PHA (Fig. 5). By contrast, the sAPPα species derived from APP695 and APP-KPI exhibited similar Con A and PHA binding.

**Table 1:** Glycosylation of sAPPα and sAPPβ in brain extracts from NDC and AD subjects

		%APP unbound to the lectin			
		sAPPα		sAPPβ	
Sample	APP specie	Con A	PHA	Con A	PHA
**NDC**	**APP695**	12.2 ± 1.7 [7.3–20.6]	6.6 ± 0.8 [4.3–9.4]	2.9 ± 0.8 [0.3–7.1]	2.6 ± 0.8 [0.2–6.8]
	**APP-KPI**	13.7 ± 2.1 [9.2–24.6]	5.7 ± 0.8 [2.8–8.1]	40.4 ± 4.6 [20.2–58.3]	37.0 ± 3.2 [22.6–51.0]
**AD**	**APP695**	7.0 ± 1.5 [2.4–12.9]	4.0 ± 0.6 [1.8–6.8]	1.2 ± 0.5 [0.1–3.7]	1.9 ± 0.3 [1.0–3.1]
	**APP-KPI**	7.7 ± 1.3 [4.0–12.4]	3.8 ± 0.6 [2.3–5.4]	57.1 ± 8.5 [37.6–89.0]	56.7 ± 9.7 [27.4–95.3]

**Fig. 6:**
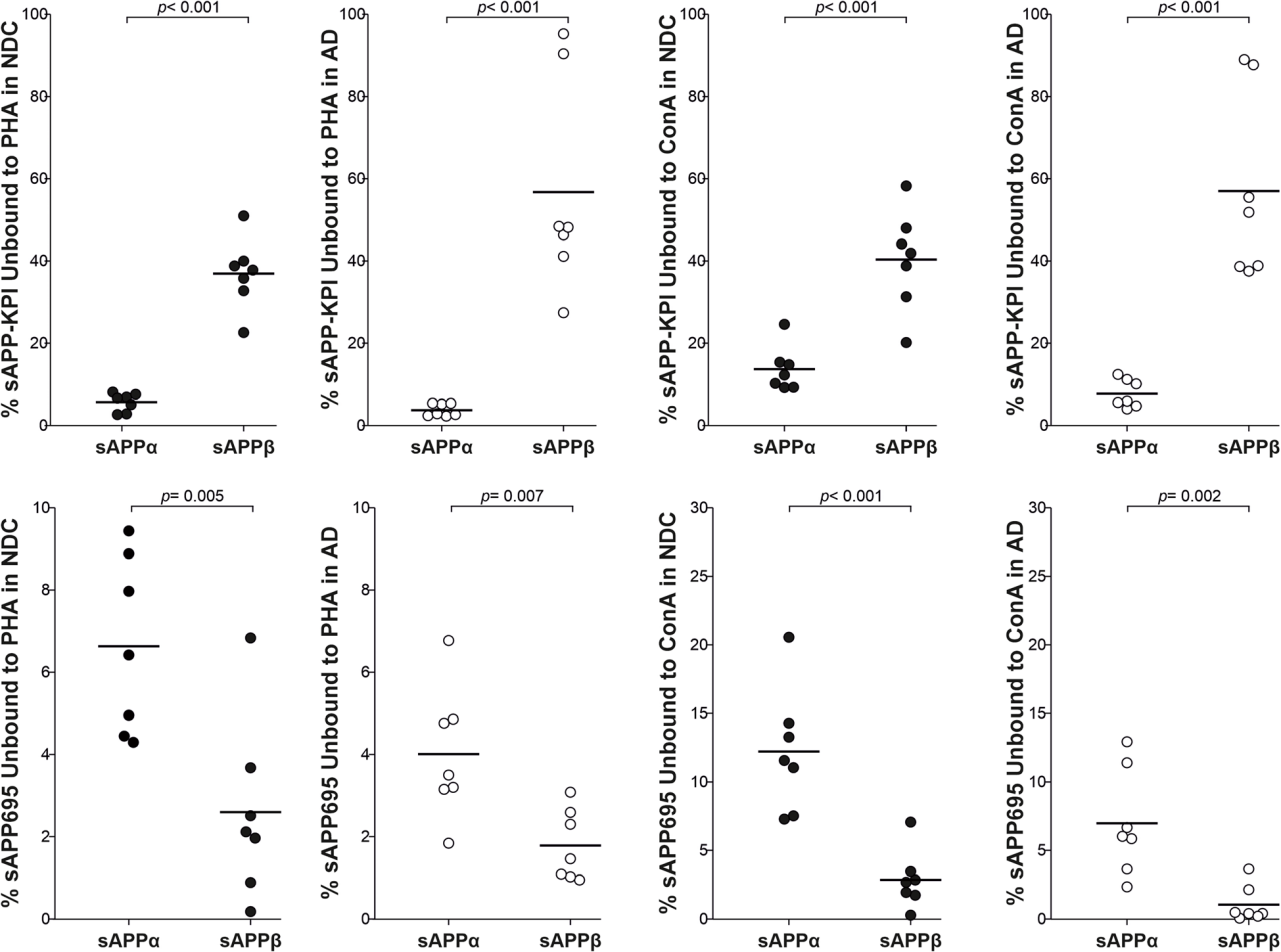
Comparison of the sAPPα and sAPPβ glycosylation in brain extracts from NDC and AD subjects. Representation of the relative (%) sAPPα and sAPPβ species derived from APP695 or APP-KPI variants from the 7 NDC and 7 AD brain extracts that did not bind to immobilized PHA and Con A lectins, as indicated in Table 1. The exact *p* values are indicated

**Fig. 7:**
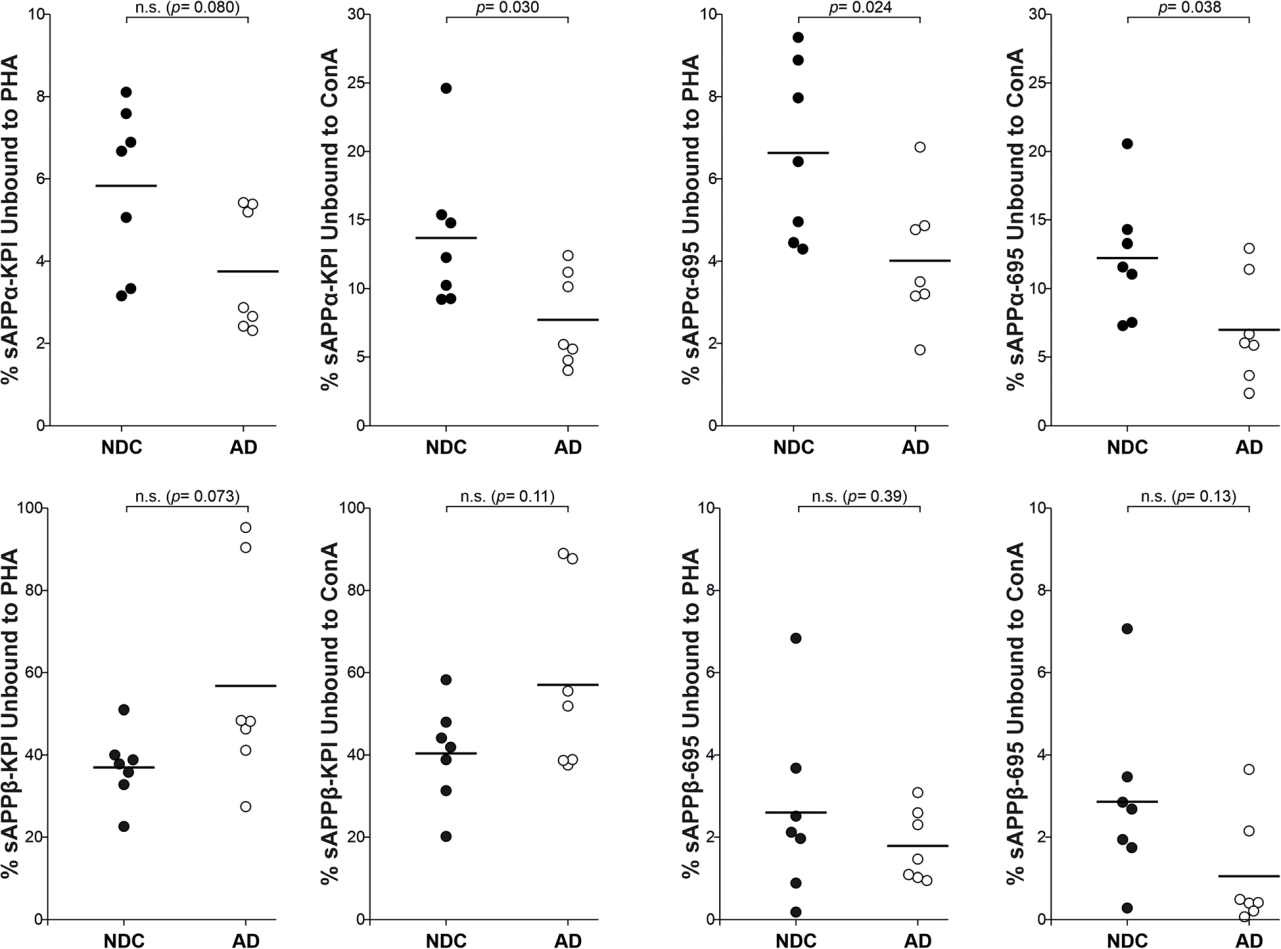
Comparison of the brain sAPP glycosylation between NDC and AD subjects. Representation of the immunoreactivity of the unbound sAPPα and sAPPβ in the 7 NDC and 7 AD brain extracts derived from APP695 and APP-KPI that did not attach to the immobilized PHA and Con A lectins (see Table 1). The exact *p* values are indicated (n.s., not significant)

The brain extracts from 7 non-demented controls (NDC) and 7 AD patients were incubated with immobilized Con A and PHA lectins. The supernatant recovered that contained the unbound protein was assayed in western blots probed with pan-specific antibodies for sAPPα and sAPPβ (see Fig. 3). The data represent the percentages (mean ± SEM) and the intervals of the unbound immunoreactivity for sAPPα and sAPPβ. These values were used to compare the differences in lectin binding between the species and groups (see Figs. 5, 6, and 7)

These data were re-analyzed to compare the lectin affinities between sAPPα and sAPPβ. Interestingly, and in accordance with the differences in molecular mass attributable to glycosylation (Fig. 2c), different patterns of sAPPα and sAPPβ glycosylation were observed for APP-NTFS derived from either APP695 or APP-KPI (Fig. 6). This suggests that glycosylation determines APP processing by either α-secretase either β-secretase, and this is a common mechanism in neurons and glia. These glycosylation differences between APP-NTFs were observed in both NDC and AD tissue.

Finally, we re-analyzed the data again, this time comparing the glycosylation pattern of each APP-NTF in AD subjects respect to NDC subjects (Fig. 7). There were significant differences in the binding of sAPPα-695 to Con A and PHA between the NDC and AD samples, and also for sAPPα-KPI binding to Con A. By contrast, there were no significant differences in AD and NDC samples when the proportion of sAPPβ that binds to Con A or PHA was compared, both for species derived from APP695 or from APP-KPI. These differences indicated that APP glycosylation is altered in the AD brain, mainly affecting the APP glycoforms processed by the non-amyloidogenic pathway.

We also examined whether Aβ could be a determining factor in the modulation of sAPPα and sAPPβ glycosylation in a cellular model that overexpress APP (APP751). CHO-PS70 cells were incubated for 2 days in the presence of 5 μM Aβ42 (added to the cells, once a day) or a scrambled Aβ42 peptide, after which the culture media were incubated with immobilized Con A and PHA lectins. The culture media from treated cells were incubated with immobilized Con A and PHA lectins. sAPPα and sAPPβ were examined in the unbound fraction by western blots, as described above for brain extracts (Table 2). Corroborating that the differences in the molecular mass observed between sAPPα and sAPPβ species (Supplemental Fig. 2C) are mainly due to differences in their glycosylation, a difference in PHA binding was observed between sAPPα and sAPPβ species in control (scrambled-treated cells). Moreover, and as occurs in the AD brain, in the cell media from Aβ42-treated cells, there was a change in the binding of sAPPα to both lectins, Con A and PHA, when compared to the control cells. The sAPPβ species from control and Aβ42-treated cells also exhibited different binding to Con A and PHA.

**Table 2:** Glycosylation of sAPPα and sAPPβ in culture media from CHO-PS70 cell treated with Aβ42

	%APP unbound to the lectin			
	sAPPα		sAPPβ	
CHO-PS70 cell media	Con A	PHA	Con A	PHA
**Control**	41.9 ± 4.8 [26.9–61.8]	62.2 ± 3.2 [51.8–74.2]	57.3 ± 5.8 [40.0–77.1]	75.0 ± 3.5^†^ [62.6–87.5]
**Aβ42**	24.7 ± 2.6* [12.9–30.0]	52.5 ± 2.4* [44.2–53.3]	28.9 ± 3.2* [39.0–16.9]	56.6 ± 3.0* [46.1–68.6]

The culture media from CHO-PS70 cells (over-expressing APP 751) treated with Aβ42 or scrambled peptide (control) were incubated with immobilized Con A and PHA lectins. The supernatant recovered containing the unbound protein was evaluated by western blots using with pan-specific antibodies for sAPPα and sAPPβ, similarly that in Table 1. Accordingly, the unbound sAPPα and sAPPβ were used to compare differences in lectin binding between groups and between sAPP species. The data represent the percentages (mean ± SEM) and the intervals of the unbound immunoreactivity for sAPPα and sAPPβ, estimated in 8 independent determinations from 2 different experiments

*Significantly different (*p* < 0.05) from the control group

^†^Significantly different (*p* < 0.05) from the sAPPα species

## Discussion

In this study, elevated APP mRNA expression was found in the brain of AD subjects when compared to control NCD individuals. Several studies have already reported increases in expression of total APP mRNA, both considered as a whole [14, 46] and when spliced APP brain isoforms are considered individually [17], particularly the APP-KPI species [37, 50, 64] or APP695 variants [28, 46]. However, as mentioned above, there is contradictory data regarding APP mRNA expression in the brain of AD patients, with several reports indicating no change or weaker expression [13, 19, 30, 66]. Technical issues associated with mRNA extraction from frozen human brain tissue, long post-mortem intervals, or other pre-analytical confounding factors could contribute to these contradictory results. In conclusion, it remains unclear if brain-specific regional and temporal changes occur in the expression of the different APP variants during AD progression.

Since APP is also found in blood cells, assessing the changes in APP mRNA expression in peripheral blood cells from AD patients has been considering an alternative. However, again the quantification of APP mRNA in peripheral blood cells has generated controversial results, with some reports indicating enhanced expression [29, 68] and others no changes or even a decrease [7, 24] in cells from AD patients. Interestingly, the changes in protein and mRNA expression in platelets from AD subjects did not parallel [16].

Brain APP protein has been analyzed in only a few studies, probably as it is difficult to interpret the complex pattern of APP variants and fragments. Interestingly, significant increases in APP synthesis and APP-CTF generation were recently demonstrated in neurons derived from AD iPSCs [44]. We previously characterized the sAPP species present in the CSF, which form heteromers involving sAPPα, sAPPβ, and also soluble full-length forms of APP. The existence of these heteromers complicates the assessment of specific sAPP species by ELISA [15]. Thus, although western blotting is less well suited to quantitative analysis than ELISA, it is a technique that avoids the interference of APP heteromers. Moreover, our approach allows the sAPPα and sAPPβ species derived from APP695 and APP-KPI to be studied separately. Here, we found a similar balance of sAPPα and sAPPβ protein, and of that between CTFα and CTFβ, in brain extracts from AD and NDC subjects. It was also notable that APP695 and APP-KPI species were only positively correlated when they derived from sAPPα but not from sAPPβ. Interestingly, despite the lack of any differences between NDC and AD patients, the ratio of APP695/APP-KPI species was associated with very different profiles of sAPPα and sAPPβ. Our results indicate that relevant amounts of sAPPβ are likely to be generated in non-neuronal cells and that their pattern of glycosylation may serve to characterize changes in AD. Nevertheless, it should be borne in mind that the normal functions of the different APP isoforms remain unclear.

The levels of APP-CTFs in the human AD brain have not been investigated thoroughly, probably because APP intracellular fragments are quickly processed by γ-secretase, and/or degraded through autophagy and the endosomal-lysosomal pathway [8, 18, 23]. Moreover, the specificity of the APP-CTF bands in western blots has been recurrently questioned [26]. Here, a parallel analysis of CHO-PS70 extracts treated with the γ-secretase inhibitor DAPT was performed to identify CTFα and CTFβ in brain extracts. The C-terminal fragments of APP are not affected by alternative splicing, and therefore, they do not serve to distinguish fragments originated from APP695 and APP-KPI. The C-terminal domains of APP are not modified by glycosylation, and thus, the same molecular masses would be expected for these fragments in cell and brain extracts. We failed to detect any significant differences in the CTFα and CTFβ in the brain of AD and NDC subjects. In summary, the elevated APP mRNA in AD brains were not paralleled by an increase in the APP fragments assayed.

We cannot rule out whether the changes in transcription are associated with changes in translation, possibly even exerting opposite effects on the final amount of protein. As such, changes in mRNA but not in protein may have a potential impact on disease progression [48]. Indeed, the protein-to-mRNA ratios for many proteins exhibit substantial tissue variability, highlighting the contribution of post-transcriptional regulation in shaping tissue-specific proteomes [21]. Although post-transcriptional regulation of APP mRNAs cannot be ruled out, the fact that the increase in APP mRNA is not mirrored by changes in the APP fragments could be associated with the rapid processing and turnover of this protein [60]. In summary, one of the few general conclusions that can probably be drawn from the different studies of the changes to APP proteolytic fragments in the brain or CSF of AD subjects is that these changes are not expected to be appropriately related to changes in APP brain expression.

The mechanisms that drive APP proteolytic processing and how each alternative pathway is regulated remain to be fully determined. Like many other transmembrane glycoproteins, typically membrane receptors, APP is a substrate of secretases [35]. The proteolytic processing of these receptors is initiated after their binding to membrane-anchored or soluble ligands. However, APP is unlikely to have “canonical” ligands that determine/regulate its processing by secretases. Anyhow, protein partners that might interact with APP at specific subcellular locations are probably influenced further by proteolytic processing driven by a particular pathway. It is well known that glycosylation influences both the subcellular localization and the protein interactions of glycoproteins. Indeed, increasing evidence indicates that glycosylation plays an important role in regulating the cleavage of APP [65]. Therefore, we explored the glycosylation status of sAPPα and sAPPβ in the AD brain through lectin binding. To our knowledge, alterations to APP glycosylation in the AD brain have yet to been thoroughly investigated.

The oligosaccharide moieties associated to glycoproteins depend on the specific enzymatic machinery in particular cell types [54] and different isoforms of the same protein may display diverse patterns of glycosylation in the same cell [55]. Indeed, we found different glycosylation patterns for sAPPβ species derived from neuronal APP695 and glial APP-KPI variants. More interestingly, we show that the sAPPα and sAPPβ originating from the same variants display differences in molecular mass that can be attributed to glycosylation and that these differences produce different lectin-binding patterns. Hence, different APP glycoforms would appear to be processed mainly through different pathways. It was previously indicated that increasing O-GlcNAcylation seems to reduce Aβ generation, and it has been considered a promising target for AD drug therapy [1, 3, 12, 33, 53, 69]. Indeed, several Aβ glycopeptides have been identified in human CSF, while the Aβ1-38/40/42 isoforms appear not to be glycosylated, with an increase of up to 2.5-fold the Tyr10 glycosylated Aβ peptides in the CSF from AD patients [25]. These data would suggest that sialylated O-glycans may influence APP processing. Hence, the large difference in molecular mass between sAPPα and sAPPβ detected here indicates that N-glycosylation influences the proteolytic processing of APP and/or its further O-glycosylation.

Our data also demonstrate differences in the glycosylation of the sAPPα species in the AD brain and that Aβ causes altered glycosylation of both sAPPα and sAPPβ, in a cellular model. Interestingly, altered glycosylation has been demonstrated for several key proteins in AD (reviewed in [32, 57]). One of the lectins studied here was PHA, which is very specific for bisecting the GlcNAc present in N-glycan structures, indicating alterations in the N-glycan profile in the AD CSF [58]. Hence, there appear to be changes in glycosylation of at least a subset of glycoproteins in the AD brain at early phases of disease progression.

Moderate changes in the glycosylation of key brain proteins may critically affect their behavior. Alterations to the glycosylation of specific glycoproteins may alter the contribution of different cell types to the protein pool, producing an imbalance in protein glycoforms, and such altered glycosylation may reflect changes in metabolism or in differentiation states. In this context, the altered glycosylation of APP in AD warrants further study, particularly as we assume that APP glycosylation determines its proteolytic processing. As such, alterations to its glycosylation may have pathophysiological consequences in terms of the generation of the diverse APP fragments.

## Conclusions

In summary, this study reveals that there is stronger expression of APP in the AD brain relative to the NDC brain and that there are important particularities in APP glycosylation that possibly affect its processing. Firstly, using lectin binding, we detected changes in glycosylation between sAPPβ species derived from APP695 and APP-KPI variants, probably reflecting the distinct cellular origin of the variants. More interestingly, differences in glycosylation pattern between sAPPα and sAPPβ suggest that glycosylation dictates whether the APP is proteolytically processed by the amyloidogenic or the non-amyloidogenic pathway. Furthermore, differences in sAPPα glycoforms between AD and non-disease control tissue indicate altered APP glycosylation in these pathological conditions. Whether altered glycosylation of APP in AD brain could explain the amyloidogenic imbalance remains to be determined.

## Supplementary information

**Additional file 1: Supplemental Figure 1.** Increased APP expression in the AD frontal cortex. Relative APP mRNA expression analyzed by *q*RT-PCR in frontal cortex tissue from NDC (*n* = 7) and AD subjects (Braak stage V-VI, n = 7). The total APP transcripts were measured using the specific TaqMan GeneExpression Assay with TaqMan PCR Master Mix. The values were calculated from relative standard curves, normalized to 18S from the same cDNA and expressed as the mean ± SEM: *p* < 0.001 relative to NDC as indicated.

**Additional file 2: Supplemental Figure 2.** Characterization of the APP-NTF and CTF fragments in human frontal cortex extracts. (**A**) To assess the identity of the sAPPα species derived from the KPI variant, two frontal cortex (Human Cx) extracts were analyzed by SDS-PAGE and probed simultaneously with a mouse anti-sAPPα combined with a rabbit anti-KPI. The fluorescence of the secondary antibodies (IRDye 800CW goat anti-rabbit, green; IRDye 680RD goat anti-mouse, red) was detected with the Odyssey CLx Infrared Imaging system (LI-COR), with simultaneous fluorescence (merge) demonstrating co-localization (yellow, arrowhead). (*) Indicates a KPI immunoreactive band that does not co-localize. No similar combination was performed for sAPPβ species since both the antibodies against sAPPβ and KPI are generated in rabbit. (**B**) The frontal cortex extracts and (**C**) the medium from CHO-PS70 (over-expressing APP751) were subjected to enzymatic deglycosylation (deGlyc), or treated with the vehicle alone as a control (Ctrl), as described in Fig. 2c, and probed simultaneously with the mouse anti-sAPPα combined with a rabbit anti-sAPPβ. The fluorescence of the secondary antibodies (IRDye 800CW goat anti-rabbit, green; IRDye 680RD goat anti-mouse, red) was detected with the Odyssey CLx for simultaneous fluorescence (yellow, merge). (**D**) Extracts from CHO-PS70 cells treated with the γ-secretase inhibitor DAPT or the vehicle alone (control, Ctrl) were probed simultaneously with the rabbit C-terminal antibody common to CTFβ and CTFα, and the rat antibody 2D8 raised against the N-terminal domain of Aβ that only detects CTFβ. The fluorescence of the secondary antibodies (IRDye 800CW goat anti-rabbit, green; IRDye 800CW goat anti-rat, red) was detected with the Odyssey CLx, with simultaneous fluorescence (merge) demonstrating co-localization (yellow). The approximate location of the molecular weight (MW) markers is shown.

**Additional file 3: Supplemental Figure 3.** Correlation of the sAβPPα and sAβPPβ species from human frontal cortex samples. (**A**) A linear regression analysis was used to assess the correlation between the sAPPα derived from the APP695 and APP-KPI variants, and between the sAPPβ derived from the APP695 and APP-KPI variants in the brain extracts from controls (NDC: closed symbols, solid lines) and AD patients (open symbols, dotted lines). (**B**) No correlation emerged from the linear regression analysis between sAPPα and sAPPβ derived from APP695 variants, or between the sAPPα and sAPPβ derived from APP-KPI variants. The linear regression coefficient (R) and *p* values for each correlation are shown (n.s., non-significant *p* value).

**Additional file 4: Supplemental Figure 4.** The APP-KPI species remain unaltered in the AD frontal cortex. Representative western blots of human frontal cortex samples from NDC (closed symbol, *n* = 7) and AD subjects (open symbol, n = 7) probed with an anti-KPI antibody. The densitometric quantification of the species attributed to the ~ 120 kDa APP-KPI band (arrowhead) is shown. Note that since the anti-KPI antibody cannot distinguish between species with a very similar molecular mass, APP-KPI levels were consider as a whole. Equivalent amounts of protein were loaded in each lane and vinculin was used as a loading control, performing calculations in duplicate. (*) Indicates a KPI immunoreactive band that does not match with bands detected with anti-sAPPα or sAPPβ (see Fig. 2b and Supplemental Fig. 2A). The comparison did not identify statistically significant differences between the AD and NDC samples (not even for the ~ 100 kDa species of uncertain identity: data not shown).

## Data Availability

All data generated or analyzed during this study are included in this published article.

## Abbreviations

APP: Amyloid precursor protein
sAPP: Soluble APP
Aβ: β-amyloid peptide
AD: Alzheimer’s disease
Con A: Lectin from *Canavalia ensiformis*
CSF: Cerebrospinal fluid
DAPT: N-[N-(3,5-difluorophenacetyl)-l-alanyl]-S-phenylglycine t-butyl ester
DMSO: Dimethyl sulfoxide
CTF: C-terminal fragment
ICD: Intracellular domain
KPI: Kunitz-type serine protease inhibitor
NDC: Non-demented controls
NTF: N-terminal fragment
PHA: Lectin from *Phaseolus vulgaris*
