# Supplementary figures and images for: Amyloid precursor protein glycosylation is altered in the brain of patients with Alzheimer’s disease
Source: Alzheimers Res Ther. 2020 Aug 12;12:96. doi: 10.1186/s13195-020-00664-9 (PMC7425076; doi:10.1186/s13195-020-00664-9)

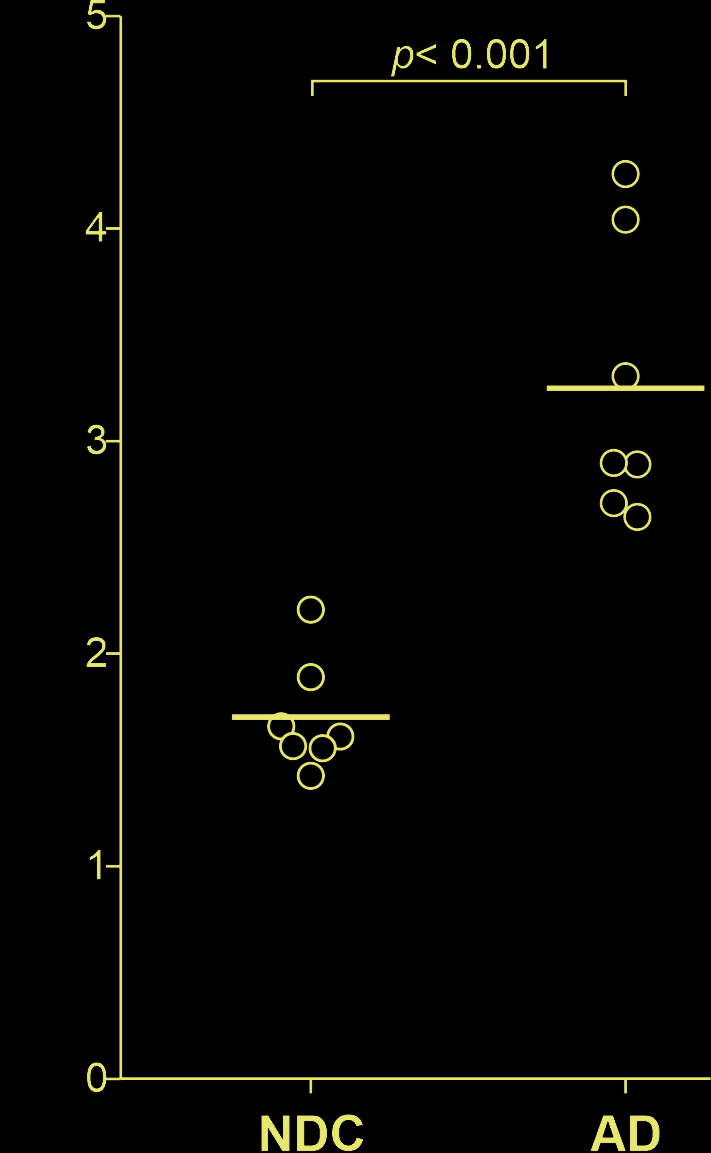

Supplement: Supplementary file 1 — Additional file 1: Supplemental Figure 1. Increased APP expression in the AD frontal cortex. Relative APP mRNA expression analyzed by qRT-PCR in frontal cortex tissue from NDC (n = 7) and AD subjects (Braak stage V-VI, n = 7). The total APP transcripts were measured using the specific TaqMan GeneExpression Assay with TaqMan PCR Master Mix. The values were calculated from relative standard curves, normalized to 18S from the same cDNA and expressed as the mean ± SEM: p < 0.001 relative to NDC as indicated. [file 13195_2020_664_MOESM1_ESM.tif]

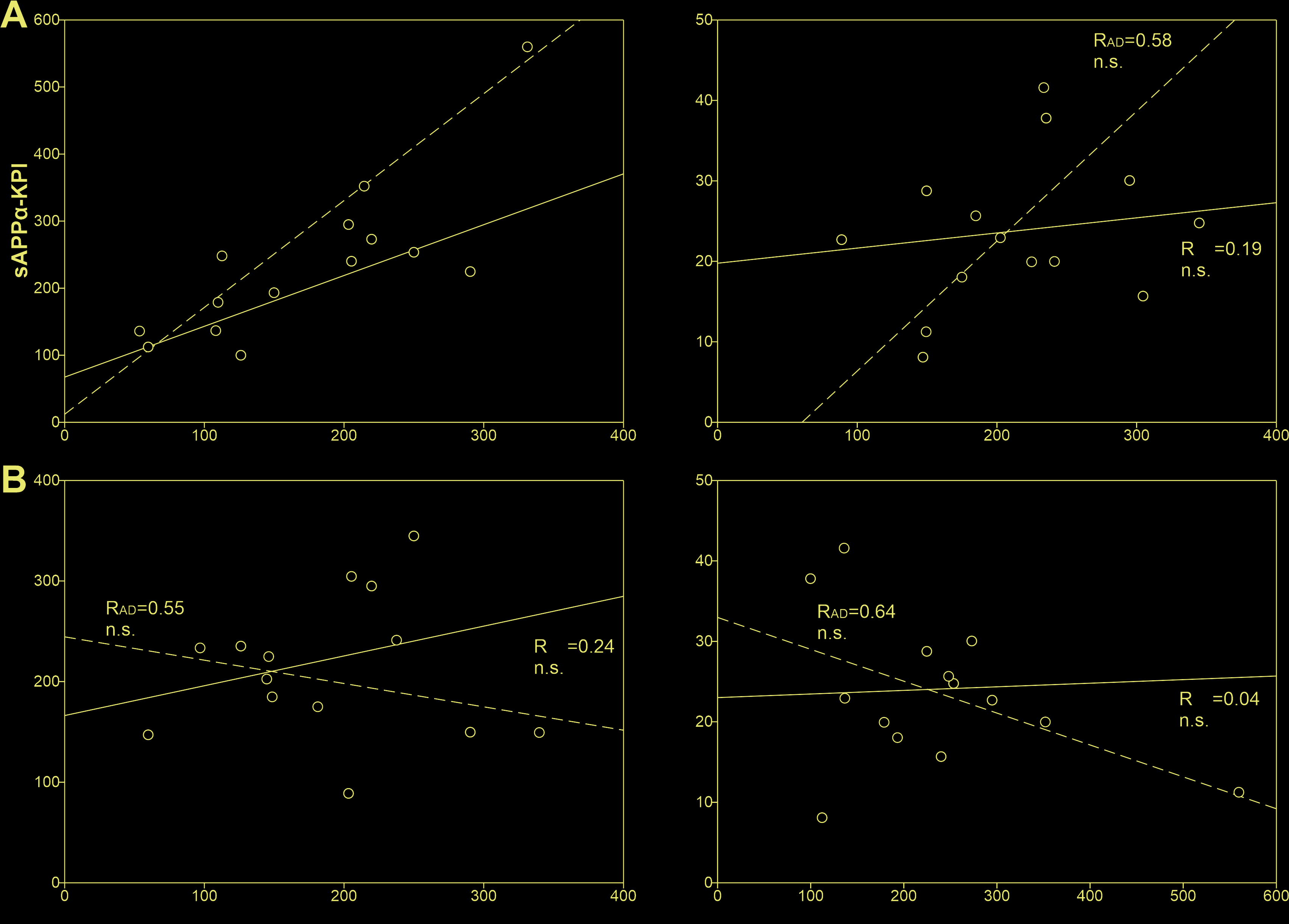

Supplement: Supplementary file 3 — Additional file 3: Supplemental Figure 3. Correlation of the sAβPPα and sAβPPβ species from human frontal cortex samples. (A) A linear regression analysis was used to assess the correlation between the sAPPα derived from the APP695 and APP-KPI variants, and between the sAPPβ derived from the APP695 and APP-KPI variants in the brain extracts from controls (NDC: closed symbols, solid lines) and AD patients (open symbols, dotted lines). (B) No correlation emerged from the linear regression analysis between sAPPα and sAPPβ derived from APP695 variants, or between the sAPPα and sAPPβ derived from APP-KPI variants. The linear regression coefficient (R) and p values for each correlation are shown (n.s., non-significant p value). [file 13195_2020_664_MOESM3_ESM.tif]

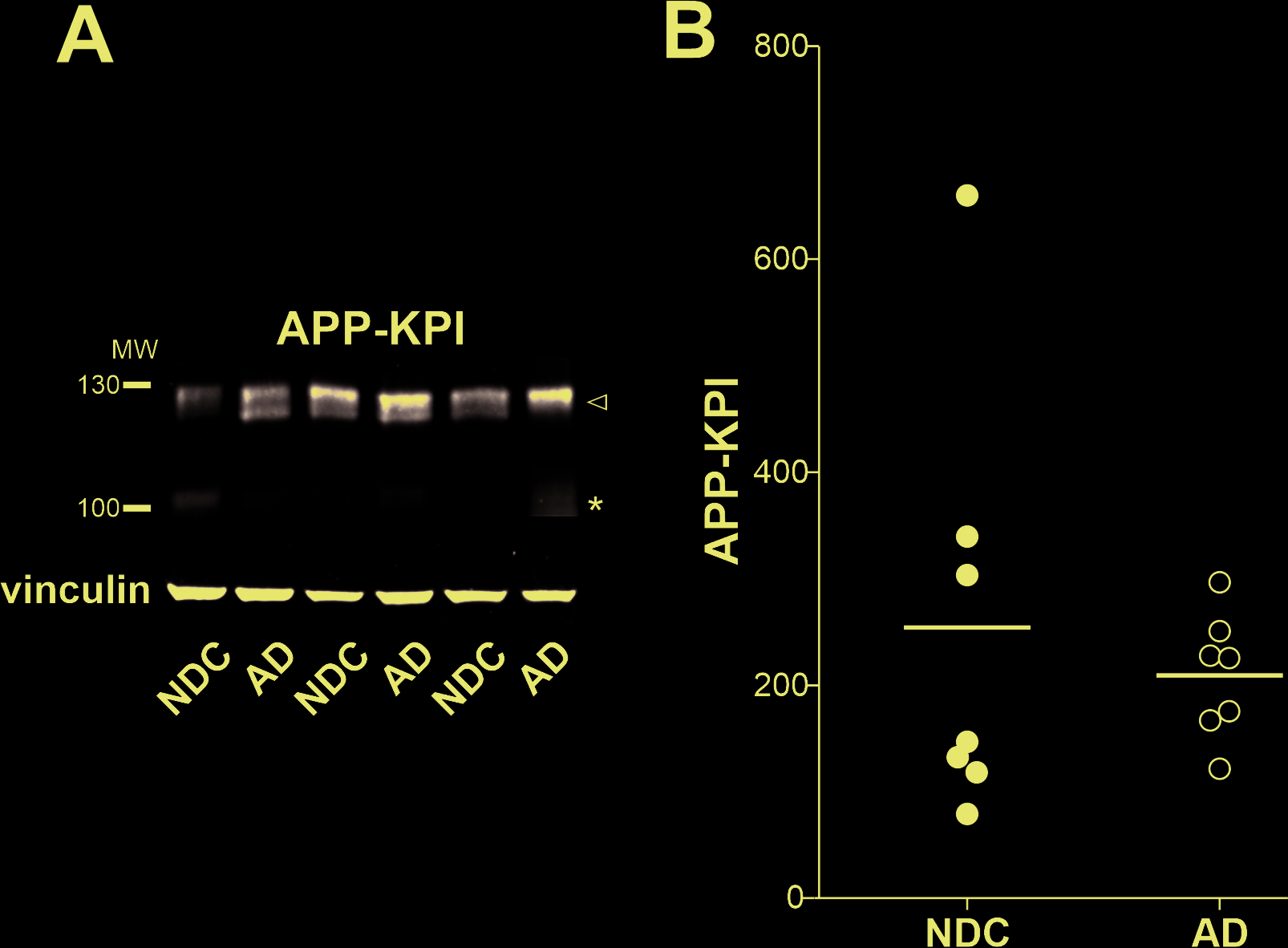

Supplement: Supplementary file 4 — Additional file 4: Supplemental Figure 4. The APP-KPI species remain unaltered in the AD frontal cortex. Representative western blots of human frontal cortex samples from NDC (closed symbol, n = 7) and AD subjects (open symbol, n = 7) probed with an anti-KPI antibody. The densitometric quantification of the species attributed to the ~ 120 kDa APP-KPI band (arrowhead) is shown. Note that since the anti-KPI antibody cannot distinguish between species with a very similar molecular mass, APP-KPI levels were consider as a whole. Equivalent amounts of protein were loaded in each lane and vinculin was used as a loading control, performing calculations in duplicate. (*) Indicates a KPI immunoreactive band that does not match with bands detected with anti-sAPPα or sAPPβ (see Fig. 2b and Supplemental Fig. 2A). The comparison did not identify statistically significant differences between the AD and NDC samples (not even for the ~ 100 kDa species of uncertain identity: data not shown). [file 13195_2020_664_MOESM4_ESM.tif]
